# Highly efficient targeted mutagenesis in one-cell mouse embryos mediated by the TALEN and CRISPR/Cas systems

**DOI:** 10.1038/srep05705

**Published:** 2014-07-16

**Authors:** Akihiro Yasue, Silvia Naomi Mitsui, Takahito Watanabe, Tetsushi Sakuma, Seiichi Oyadomari, Takashi Yamamoto, Sumihare Noji, Taro Mito, Eiji Tanaka

**Affiliations:** 1 Department of Orthodontics and Dentofacial Orthopedics, Institute of Health Biosciences, The University of Tokushima Graduate School, 3-18-15 Kuramoto-cho, Tokushima 770-8504, Japan; 2 Department of Life Systems, Institute of Technology and Science, The University of Tokushima, 2-1 Minami-Jyosanjima-cho, Tokushima 770-8506, Japan; 3 Department of Mathematical and Life Sciences, Graduate School of Science, Hiroshima University, 1-3-1 Kagamiyama, Higashi-Hiroshima, Hiroshima 739-8526, Japan; 4 Division of Molecular Biology, Institute for Genome Research, The University of Tokushima, 3-18-15 Kuramoto-cho, Tokushima 770-8503, Japan

## Abstract

Since the establishment of embryonic stem (ES) cell lines, the combined use of gene targeting with homologous recombination has aided in elucidating the functions of various genes. However, the ES cell technique is inefficient and time-consuming. Recently, two new gene-targeting technologies have been developed: the transcription activator-like effector nuclease (TALEN) system, and the clustered regularly interspaced short palindromic repeat (CRISPR)/CRISPR-associated protein (Cas) system. In addition to aiding researchers in solving conventional problems, these technologies can be used to induce site-specific mutations in various species for which ES cells have not been established. Here, by targeting the Fgf10 gene through RNA microinjection in one-cell mouse embryos with the TALEN and CRISPR/Cas systems, we produced the known limb-defect phenotypes of Fgf10-deficient embryos at the F0 generation. Compared to the TALEN system, the CRISPR/Cas system induced the limb-defect phenotypes with a strikingly higher efficiency. Our results demonstrate that although both gene-targeting technologies are useful, the CRISPR/Cas system more effectively elicits single-step biallelic mutations in mice.

Methodologies for disrupting the genome can be used to further our understanding of biological mechanisms. *Forward genetic approaches*, such as invoking the mismatch repair system with alkylating agents (*e.g.* ethylmethanesulfonate and ethylnitrosourea) and inducing mutagenesis by ultraviolet trimethylpsoralen or chromosomal transposon insertion, are mainly used to analyse the precise function of genes. *Reverse genetic analyses* of mice and rats have become possible through the development of embryonic stem (ES) cells. However, although well-developed and extensively used, gene-targeting technology using ES cells is costly and time-consuming^1^. Therefore, researchers are seeking to apply new genome-editing tools to mice as the next generation of genome-modification technologies. Recently, several powerful techniques, including zinc finger nucleases (ZFNs), transcription activator-like (TAL) effector nucleases (TALENs) and the clustered regularly interspaced short palindromic repeat (CRISPR)/CRISPR-associated protein (Cas) system, have been developed and used to edit the genomes of cultured cells and various organisms^2^,^3^,^4^.

TALENs consist of an engineered array of TAL effector repeats fused to the FokI cleavage domain. By binding to specific sequences on opposing DNA strands, TALENs introduce double-strand breaks (DSBs) into the target gene^5^. In mammalian zygotes, TALEN-mediated gene targeting was first performed in rats^6^ *via* RNA microinjection into one-cell embryos and, later, in mice^7^,^8^,^9^. CRISPR/Cas is an RNA-based adaptive immune system developed by bacteria and archaea to cleave foreign nucleic acids (*e.g.* viral genomes and plasmids)^10^. The complex of a CRISPR RNA (crRNA) and a trans-activating crRNA (tracrRNA) was shown to guide the Cas9 endonuclease to a specific DNA target sequence to generate DSBs^11^. An engineered single guide RNA (sgRNA), consisting of crRNA fused with tracrRNA, also directed sequence-specific Cas9-mediated cleavage^10^. CRISPR/Cas has been applied to cultured human cells^12^,^13^, flies, zebrafish, mice and rats for genome-editing purposes^14^,^15^,^16^,^17^. Gene targeting with CRISPR/Cas in one-cell mouse and rat embryos allowed the highly efficient production of heritable single or multiple gene mutations at the F0 generation^16^,^17^.

Previous studies have demonstrated greater efficiency of gene targeting with CRISPR/Cas compared to the TALEN system in zebrafish and mice^6^,^7^,^16^,^17^,^18^. However, to our knowledge, no study has described the differences in effects on the same target sequence or gene between these two genome-modifying systems. Here, we report the highly efficient disruption of the Fgf10 gene in mice *via* the TALEN and CRISPR/Cas systems.

## Results

### Mutagenesis of an endogenous gene using TALENs

Fgf10 is a member of the fibroblast growth factor family. It plays an essential role in mesenchymal-epithelial interactions for the proper development of many organs. Fgf10-deficient mice show an obvious phenotype of complete limb deficiency^19^. Thus, the effects of DSBs in Fgf10 can be directly observed *via* this easily identifiable morphological phenotype. Accordingly, we chose mouse Fgf10 as the target gene for our study.

**Figure 1:**
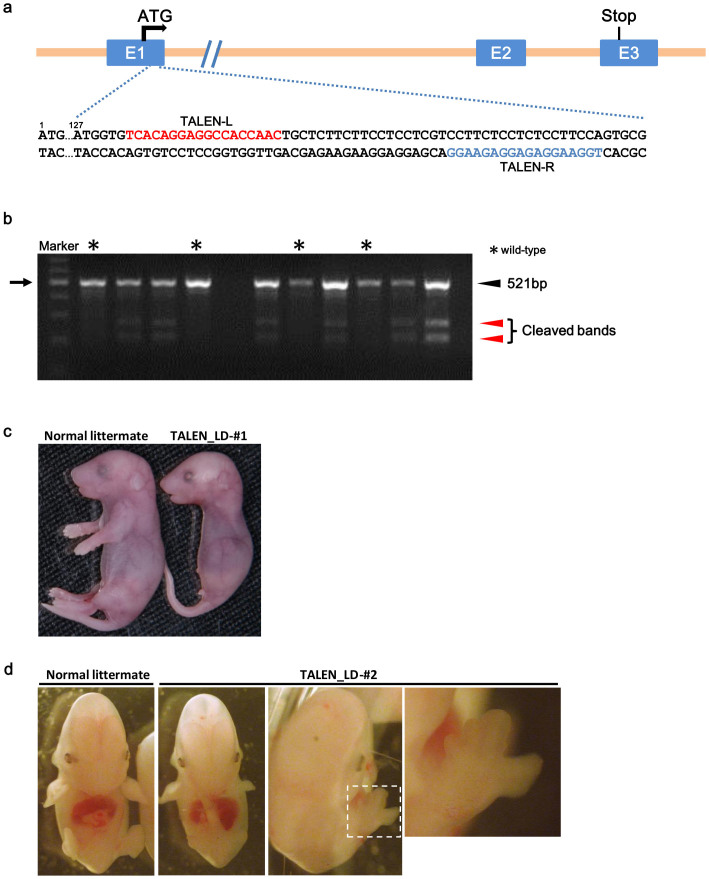
Design and application of TALENs targeted to mouse Fgf10. (a) Fgf10 genomic structure and target sequences of TALENs in the mouse Fgf10 locus. Red and blue sequences indicate the left and right binding sites of Fgf10-TALEN, respectively. (b) Example of the Surveyor endonuclease assay shows cleavage of the PCR amplicons from embryos injected with TALEN mRNAs. Asterisks are the lanes for embryos possessing undisrupted target sequences in the genome. Black arrowhead indicates fragments corresponding to predicted cleavage of the 521-bp amplicon. Red arrowheads indicate products generated from Surveyor nuclease assays. Arrow indicates the 500-bp size marker. (c) F0 embryo with limb defects after injection with TALEN mRNAs (E18.5). Complete limb deficiency was observed in the Fgf10-deficient embryo TALEN_LD-#1. (d) F0 embryo with limb defects after injection with TALEN mRNAs (E13.5). TALEN_LD-#2 showed an abnormal limb development only in the right forelimb (magnified in the right panels).

The Fgf10 gene consists of three exons. Transposagen Biopharmaceuticals, Inc. designed a TALEN pair with a 19-bp spacer in exon 1 (Fig. 1a). TALEN mRNAs produced by *in vitro* transcription were microinjected at different concentrations (10, 20 or 50 ng/μl) into the cytoplasm or pronuclei of fertilized mouse eggs. After an overnight *in vitro* culture of the injected oocytes, two-cell embryos were transferred into pseudo-pregnant female mice. The resulting embryos or pups were assayed for alteration of the TALEN-targeted Fgf10 locus by direct sequencing and the Surveyor (Cel-I) endonuclease assay (Fig. 1b). As shown in Table 1, when TALEN mRNAs at 50, 20 and 10 ng/μl were injected into the cytoplasm, 48% (13/27), 26% (11/43) and 27% (16/59) of embryos, respectively, exhibited non-homologous end joining (NHEJ). When mRNAs at 20 and 10 ng/μl were injected into pronuclei, the Fgf10 gene was modified in 13% (1/8) and 21% (4/19) of embryos, respectively. When TALEN mRNAs at 50 ng/μl were injected into the cytoplasm, 47% (15/32) of pups showed Fgf10 gene modification.

**Figure 2:**
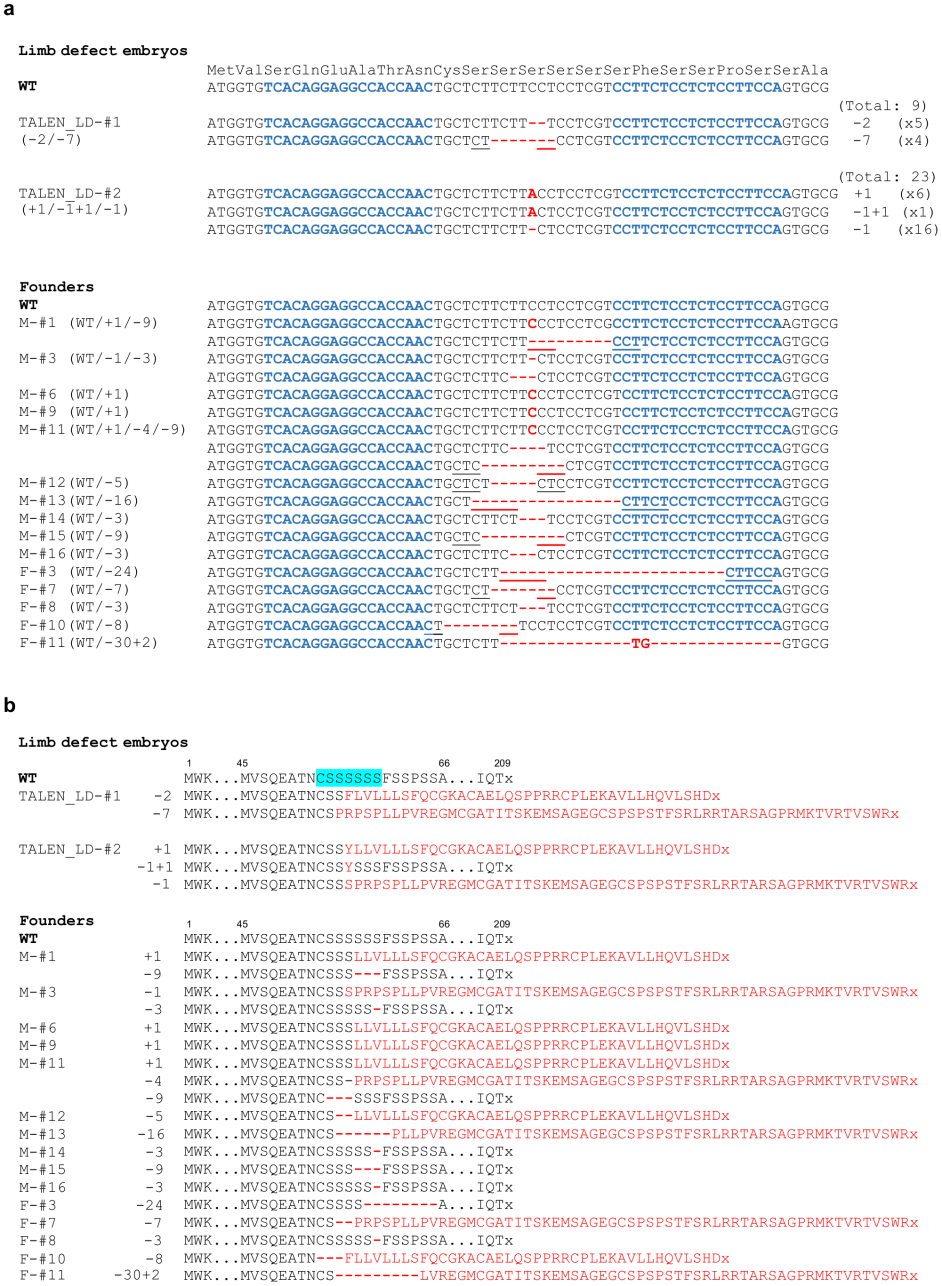
Sequence information of Fgf10 mutant alleles produced by TALEN. (a) Sequences obtained from embryos with limb defects or from founder animals generated by microinjection of TALEN mRNAs. DNA sequences to which the TALEN monomers were designed are shown in blue text. Nucleotide mutations (deletions and insertions) are shown in red text. Microhomologous sequences adjacent to the breakpoint are underlined. For F0 embryos with limb defects, TA clones of the PCR products amplified from the genomic DNA were analysed. The total number of examined clones and the number of each genotype are listed at the right side. (b) Wild-type sequences of the Fgf10 protein are shown on the top. Amino acids corresponding to spacer region are highlighted in blue. Predicted consequences of the mutation on the amino acid sequences are highlighted in red. Stop codons are shown as x.

Two F0 embryos with limb defects were obtained by microinjection of TALEN mRNAs (Fig. 1c, d). Sequence analysis was performed of these two embryos and the founder lines (Fig. 2a, b). The embryo TALEN_LD-#1 possessed limb defects and biallelic null mutations (−2/−7). TALEN_LD-#2 exhibited non-uniform right forelimb development and carried mosaic alleles (+1/−1+1/−1). Some mosaic founders (#1, 3, 11) were occasionally observed (Fig. 2). Flanking of the DSBs by short sequence repeats was previously reported in both TALEN- and CRISPR/Cas-induced mutations in mice and rats. This same effect was likely responsible for the preferential generation of the observed alleles^6^,^16^,^20^. In TALEN-induced mutations, several alleles (*e.g.* +1 of C, −1, −3 of two places, −7, −9) were repeatedly recovered in independently derived mice. Several alleles (*e.g.* −5, −7, −8, −9, −16, −24) were considered to be formed by microhomology-mediated end joining (MMEJ)^21^,^22^.

### Germline transmission and generation of homozygous knockout mice

We bred several F0 mice or their offspring with wild-type or mutant mice to confirm the germline transmission of each allele found in mosaic animals and the disruption of gene function. We assayed the resulting embryos for each allele present in their parents by sequence analysis. All of the examined TALEN-modified alleles were confirmed to be transmitted through the germline. However, a homozygous mutant with −9/−30+2 alleles (offspring of WT/+1/−9 [M1] × WT/−30+2 [F11]) did not show any limb-defect phenotype (Table 2). Expected embryos presenting limb-defect phenotypes were obtained for the offspring of WT/−5 × WT/−1 and WT/−1 × WT/−1.

### Gene knockout using CRISPR/Cas

**Figure 3:**
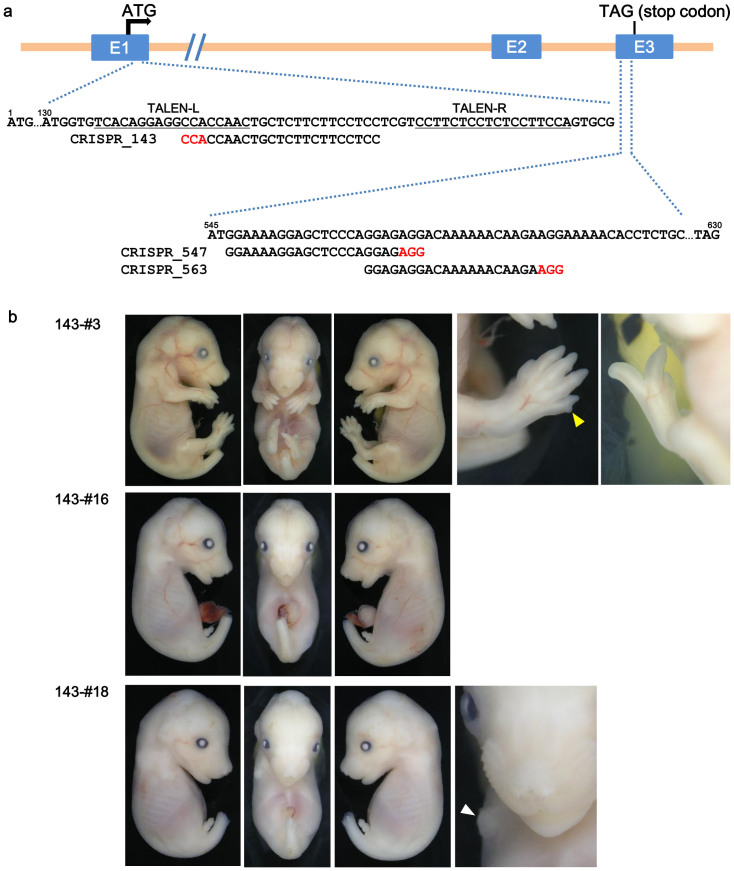
Fgf10 genomic structure and target sequences of CRISPR/Cas system, and examples of embryos produced by CRISPR_143. (a) Fgf10 genomic structure and target sequences of CRISPR_143, 547 and 563 in the mouse Fgf10 locus. Protospacer adjacent motif (PAM) sequences are highlighted in red. TALEN-binding sites described in Fig. 1 are underlined. (b) Examples of embryos with limb defects (143-#3, #16 and #18) are shown (E16.5). Embryo 143-#3 shows mosaic phenotypes in both hindlimbs. The yellow arrowhead indicates an extra digit in the right forelimb. Embryo 143-#16 with only -14 allele shows the complete limb-deficiency phenotype typical of Fgf10-deficient embryos. Embryo 143-#18 exhibits a small prominence at the right forelimb area (white arrowhead).

Next, we selected the same region to the TALEN target site (CRISPR_143) for gene knockout with the CRISPR/Cas system (Fig. 3a). Cas9 mRNAs and sgRNA produced by *in vitro* transcription were microinjected into the cytoplasm of fertilized mouse eggs. After the injected oocytes were cultured overnight *in vitro*, two-cell embryos were transferred into pseudo-pregnant female mice. Embryos were isolated between embryonic day 14.5 (E14.5) and E17.0. Of the 25 embryos injected with CRISPR_143, 14 embryos (56%) at F0 showed limb defects (Table 3). Some embryos had various limb-defect phenotypes (Fig. 3b). The embryos were assayed for alteration of the CRISPR-targeted Fgf10 loci by direct sequencing and Cel-I endonuclease. NHEJ events were detected in 24 embryos (96%, Table 3).

**Figure 4:**
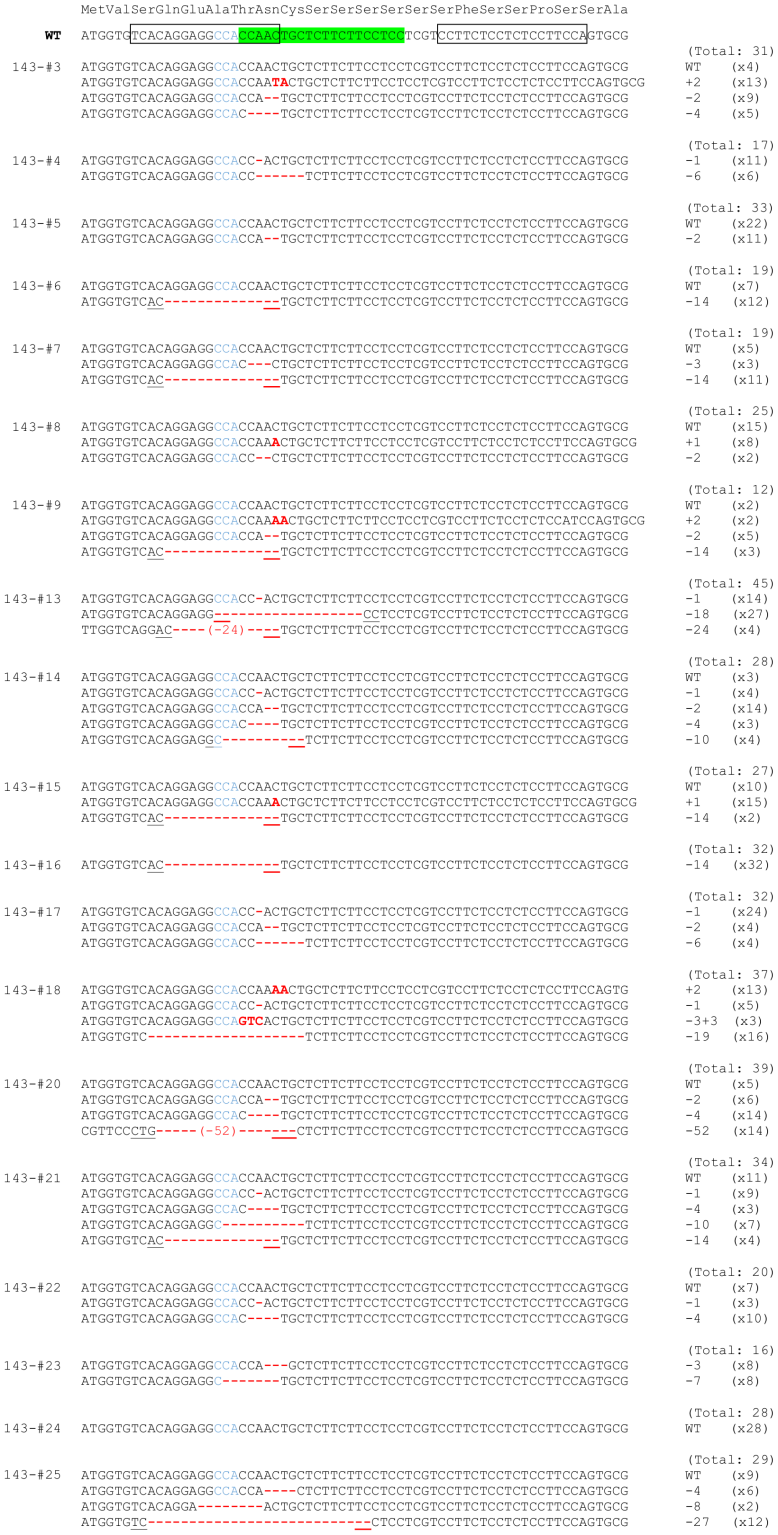
Sequence information of Fgf10 mutant alleles produced by CRISPR_143 sgRNA and Cas9 mRNAs. For F0 embryos, TA clones of the PCR products amplified from each embryo were analysed by DNA sequencing. Total number of clones examined and number of each genotype are listed at the right side. Protospacer and PAM sequences are highlighted in green and shown in blue text, respectively. Boxed sequences represent TALEN-binding sites described in Fig. 1. Microhomologous sequences adjacent to the breakpoint are underlined.

Fig. 4 reports the DNA sequences of the obtained embryos, some of which showed mosaic phenotypes (Figs. 3b, 4), including 143-#3 (WT/+2/−2/−4), #18 (+2/−1/−3+3/−19), #20 (WT/−2/−4/−52) and #21 (WT/−1/−4/−10/−14). Embryos 143-#4–#8, #13, #14 and #22–#25 were visually normal; however, wild-type alleles were not detected for embryos 143-#4 (−1/−6), #13 (−1/−18/−24) or #23 (−3/−7) (Fig. 4). Embryo 143-#16, in which all 32 clones were 14-nucleotide deletions, exhibited the complete limb-deficiency phenotype (Figs. 3b, 4). Several alleles (*e.g.* −1, −2, −4, −10, −14) were commonly recovered. Six alleles (*e.g.* −10, −14, −18, −24, −27, −52) were considered to be formed by MMEJ.

Previous studies identified causative mutations of aplasia of the lacrimal and salivary glands (ALSG) and lacrimo-auriculo-dento-digital (LADD) syndrome, which are overlapping clinical entities, in the *FGF10* gene^23^,^24^,^25^. These mutations included a missense mutation in exon 3, nonsense mutations in exons 2 and 3, and a substitution of the terminal nucleotide of intron 2, leading to loss of the splice acceptor site of exon 3, affecting the C-terminal region. Next, we sought to verify the importance of exon 3 for protein function.

We designed the CRISPR_547 and CRISPR_563 constructs in exon3 of the Fgf10 gene (Fig. 3a). Cas9 mRNAs and sgRNA were microinjected and egg transfer was performed as described for CRISPR_143. When the CRISPR_547 and CRISPR_563 constructs were used, 94% (17/18) and 58% (18/31) of the F0-generation embryos, respectively, had altered limb development (Table 3). Some embryos exhibited various limb-defect phenotypes (Fig. S1). For the CRISPR_547 construct with 10 ng/μl Cas9 mRNA and 1 or 0.1 ng/μl sgRNAs, 100% (4/4) or 93% (13/14) of embryos, respectively, showed limb-defect phenotypes, compared to 91% (10/11) or 40% (8/20), respectively, for the CRISPR_563 construct (Table 3).

**Figure 5:**
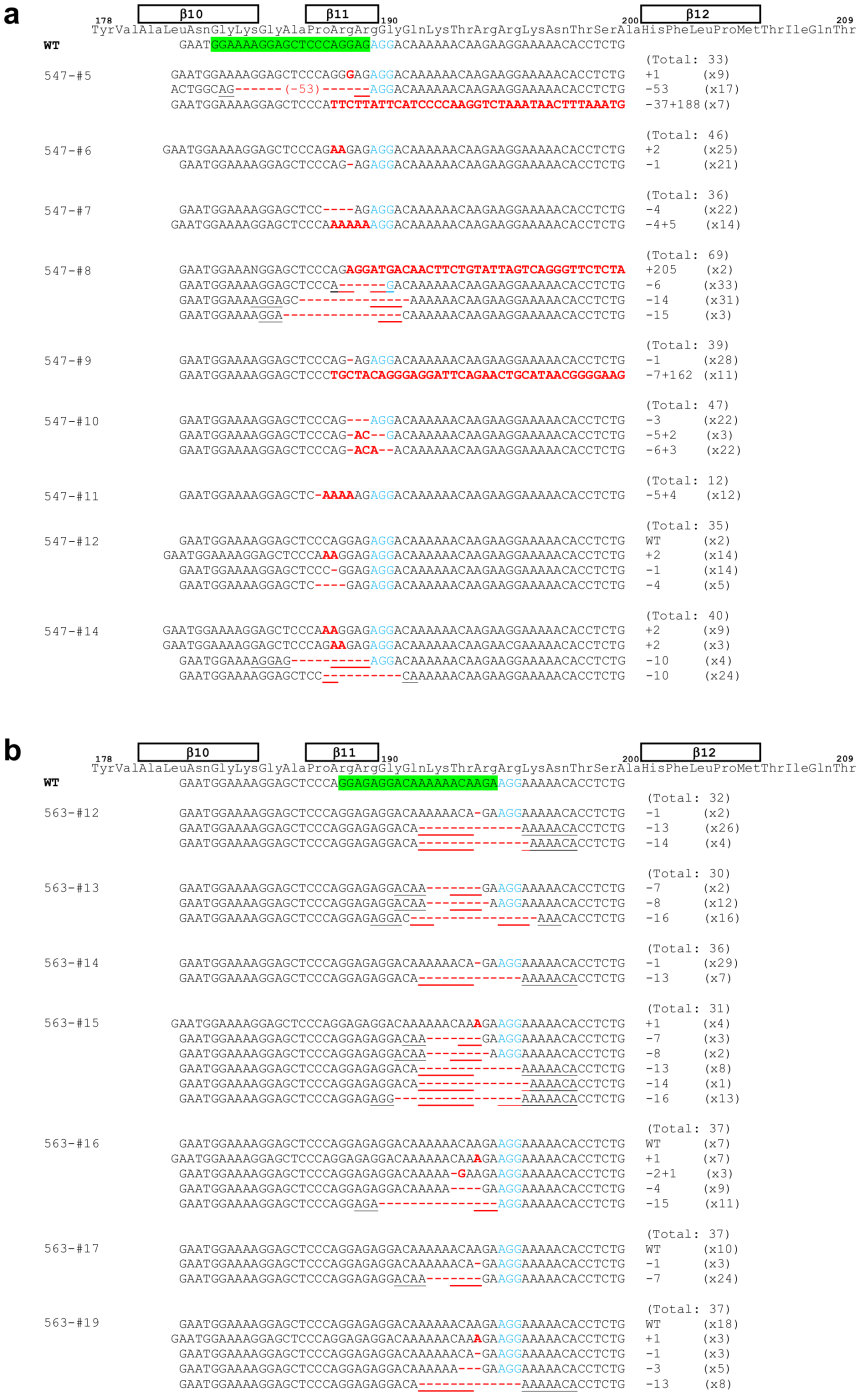
Sequence information of Fgf10 mutant alleles produced by CRISPR_547 and 563 sgRNA and Cas9 mRNAs. For F0 embryos with limb defects, TA clones of the PCR products amplified from each embryo were analysed by DNA sequencing. The total number of clones examined and the number of each genotype are listed at the right side. Protospacer and PAM sequences are highlighted in green and shown in blue text, respectively. Microhomologous sequences adjacent to the breakpoint are underlined. Positions of β-strands are shown above the corresponding amino acid sequences (β10–β12).

When the CRISPR_547 construct was used, the complete limb-deficiency phenotype was observed in embryos 547-#5–#12 and #14. Embryos 547-#5–#11 and #14 possessed biallelic or mosaic mutations without wild-type alleles. In embryos 547-#8 and #10, half or all of the detected alleles were in-frame indels, which are evenly divisible by three (Figs. 5 and S1). Embryo 547-#12 had eyelids, which should be lost in Fgf10^−/−^ mice (Figs. 5 and S1), and only 2/35 clones had the wild-type allele. Some alleles (*e.g.* +2, −1) were occasionally recovered. Several alleles (*e.g.* −6, −10, −14, −15, −53) were considered to be formed by MMEJ.

For CRISPR_563, embryos 563-#12–#15 visually exhibited the complete limb-deficiency phenotype. These embryos possessed mosaic mutations without wild-type alleles or in-frame indels. Embryos 563-#16, #17 and #19, which possessed wild-type alleles, exhibited non-uniform limb-defect phenotypes (Fig. 5, S1). Some alleles (*e.g.* +1, −1, −7, −8, −13) were occasionally recovered. Several alleles (*e.g.* −7, −8, −13, −14, −15, −16) were considered to be formed by MMEJ.

## Discussion

The TALEN and CRISPR/Cas systems have recently become more popular for genome editing than ZFNs, probably because of the flexibility and easy preparation of the former constructs. ZFN-induced mutations have an efficiency of up to ~7.4% (20–75% of live births)^26^,^27^,^28^. Widely different mutagenic efficiencies have been reported for TALENs, probably due to the numerous methods of constructing TALENs harbouring different TAL effector scaffolds and repeat variants. TALEN efficiencies of up to 15% have been reported for some targets, including fragile-site genes (*e.g.* with AT-rich and dinucleotide–repeat regions)^29^, the repeat-rich Y chromosome^30^, and microRNA genes^31^. Efficiencies of 40–100% were reported for TALEN-induced mutagenesis^32^,^33^,^34^. High efficiencies have been reported for targeted gene disruption with CRISPR/Cas (*e.g.* >75% of founders with mutations^17^ and a biallelic mutation induction efficiency of ≤50%^16^). Although the TALEN and CRISPR/Cas systems have been used to disrupt the genome in mice^7^,^8^,^16^,^17^, to the best of our knowledge, this is the first report to show disruption of the same gene by both methods. Fgf10 was selected as the marker gene because of its obvious limb-deficiency phenotype in the knockout embryo^19^.

Previous studies compared the efficiencies of indel mutagenesis by TALEN and CRISPR/Cas in mammalian cells and zebrafish. Although CRISPR/Cas elicited a higher mutagenic activity (up to ~29%) than TALEN (up to ~4.5%) in mammalian cells^12^, TALEN showed superior performance in zebrafish (Cas9/gRNA: ~3–70%, TALEN: ~5–100%)^18^. In the present study, when the TALEN system was used, the efficiency of NHEJ mutations was as high as ~50%, and the frequency of F0 embryos with limb defects was as high as ~10%. We, then, evaluated the capacity of CRISPR/Cas system to disrupt genes in mice, by selecting the same region to the TALEN target site (CRISPR_143). The efficiency of TALEN-induced NHEJ events ranged 27–48%, whereas the CRISPR_143-induced mutation rate exceeded 90%. DSBs are efficiently repaired by MMEJ and homologous recombination. MMEJ is a Ku-independent end-joining method mediated by base pairing between microhomologous sequences of 5–25 nucleotides^21^. In this study, although both TALEN and CIRSPR/Cas induced many types of indels in the same region, they did not produce the same indels. Because the PAM sequence recognized by Cas9 nuclease is slightly separated from the TALEN spacer region, the short sequence repeats used for DSB repair might be different between them.

For the limb phenotypes, embryos injected with sgRNA (CRISPR_143) and Cas9 mRNA showed higher frequencies of limb-defect phenotypes (up to ~60%) compared to embryos injected with TALEN mRNAs (up to ~10%) at the F0 generation. Moreover, the CRISPR/Cas system may remain active until later in embryogenesis than the TALEN system from the variety of mutant alleles detected in each mosaic animal. Mutant alleles induced by either system reportedly transmit to the next generation at similar rates^7^,^8^,^16^,^17^. Although we did not examine the germline transmission of CRISPR/Cas-induced mutant alleles, all of the examined TALEN-induced alleles were transmitted to the next generation.

Gene targeting method using TALEN and CRISPR/Cas system is spreading explosively, however, the indels which are multiple of three need be taken care of. Embryos 143-#4 (−1/−6), #13 (−1/−18/−24) and #23 (−3/−7) appeared normal without possessing wild-type alleles. An embryo with the −9/−30+2 alleles also exhibited a normal phenotype (Table 2). From the sequence homology, the crystal structure of human FGF10 is anticipated to adopt a β-trefoil fold consisting of 12 β-strands (β1–β12) after the N-terminal 3_10_ helix^35^. The region upstream of the 3_10_ helix was selected as the target site for the TALEN system. Therefore, in-frame indels upstream of the β-trefoil fold structure, which is the target site of TALEN and CRISPR_143, may not affect the FGF10 protein function.

To create gene-knockout animals, the exon including the start codon or a common exon among splice variants is generally chosen as the target locus. Sekine *et al*. generated Fgf10-deficient mice by replacing the exon encoding the ATG translational start site with the neomycin cassette^19^. Thus far, it has not been realistic to identify the functional domain of a protein *in vivo* by deletion analysis, although this approach is often performed for cultured cells. In the present study, we also targeted exon 3 in the mouse Fgf10 gene, which corresponds to several reported human ALSG mutations. The target site overlaps with the β11 strand of the FGF10 protein for CRISPR_547 and is located between the β11 and β12 strands of CRISPR_563. Lys-196 (next to the protospacer adjacent motif [PAM]) of CRISPR_563) and His-201 (in the β12 strand) are important residues for the secondary ligand-receptor interaction in the FGF10-FGFR2b complex^36^. Termination at Arg-194 next to the PAM sequence of CRISPR_563 leads to ALSG^23^. A histidine-to-proline substitution downstream of the β12 strand was not related to ALSG^37^, although the histidine residue is not conserved in mouse FGF10. CRISPR_547 and 563 produced embryos with limb-defect phenotypes at a strikingly higher rate (up to ~100%) than TALEN (up to ~10%; Table 3). Moreover, the complete limb-deficiency phenotype was observed for embryo 547-#8, which possessed in-frame indels in almost half of the examined clones, and embryo 547-#10, which possessed only in-frame indels. These previous findings confirm the *in vivo* importance of the β11-β12 loop at the C-terminus of FGF10.

Mosaicism and abnormal limb development were occasionally observed in embryos treated by TALENs or CRISPR/Cas (Figs. 1, 3, and S1). Embryos with mosaic phenotypes (*e.g.* TALEN_LD-#2, CRISPR_143-#3, #15, #18, #20, #21, CRISPR_563-#16, #17 and #19) carried frame-shift alleles, wild-type alleles or in-frame indels (Fig. 2, 4, 5). Because the target sites of TALEN and CRISPR_143 are upstream of the 3_10_ helix and β-strands, in-frame indels of the −1+1 allele in TALEN_LD-#2 (1/23 clones) and the −3+3 allele in CRISPR_143-#18 (3/37 clones) did not affect the functional structure of the FGF10 protein, which might lead to mosaic phenotypes without wild-type alleles. However, embryos 547-#8 and #10, in which half or all of the clones were in-frame indels, exhibited the complete limb-deficiency phenotype. These observations suggest that selecting a target site in important domains may be important for effectively disrupting the gene function.

The CRISPR/Cas system has some advantages for genome editing, including easier preparation of the constructs compared to TALENs. However, the off-target cleavage rate may be high for short or single target sequences. A high frequency of off-target mutagenesis for the CRISPR/Cas system was reported in human cultured cells^38^,^39^ and some *in vivo* mouse analyses^16^,^40^,^41^,^42^. Using Cas9 nickase would efficiently reduce the damage at known off-target sites^43^,^44^. Although we did not investigate the off-target mutagenesis rate, we efficiently obtained the expected phenotypes at the F0 generation through several different target sites in the Fgf10 gene, especially by the CRISPR/Cas system.

In conclusion, we have described the differences in effects on the same target region or gene between the TALEN and CRISPR/Cas systems. The CRISPR/Cas system showed extremely high rates of NHEJ events in multiple alleles and, similar to the TALEN system, is an excellent tool to accelerate functional genomic research in mice.

## Methods

### Animals

All animal care and experiments were carried out in accordance with the Guidelines for Animal Experiments of the University of Tokushima, and were approved by the Ethics Committee of the University of Tokushima for Animal Research (approval number: 10110).

### Production of TALEN mRNA

TALEN plasmids targeting the mouse Fgf10 exon1 sequences were synthesized by Transposagen Biopharmaceuticals, Inc. (Lexington, KY). To prepare mRNA for Fgf10, TALEN plasmid linearization with StuI and phenol-chloroform extraction were performed by standard methods. The mRNAs were generated *in vitro* with the mMESSAGE mMACHINE T7 ULTRA Kit (Ambion, Austin, TX), according to the manufacturer's instructions. After ethanol precipitation, mRNAs were suspended in an appropriate volume of RNase-free water to achieve final concentrations of 10, 20 and 50 ng/μl.

### Production of Cas9 mRNA and sgRNA

The Cas9 plasmid (pMLM3613) was purchased from Addgene (Cambridge, MA). For sgRNA production, two different forward and reverse primers for target sequences of Fgf10 were annealed and cloned into the BsaI site of the pDR274-encoding sgRNA. Cas9 mRNA and sgRNAs were transcribed using PmeI-digested Cas9 and DraI-digested gRNA expression vectors as templates by the mMESSAGE mMACHINE T7 ULTRA Kit. After transcription was complete, DNase I treatment was performed according to the manufacturer's instructions. Cas9-encoding mRNA and sgRNA were purified by LiCl and isopropanol precipitation. They were suspended in an appropriate volume of RNase-free water. RNA concentrations for microinjection were estimated from the bands and DNA markers.

### RNA microinjection into one-cell mouse embryos

TALEN mRNAs were microinjected into the cytoplasm or pronuclei of fertilized eggs derived from superovulated BDF1 (C57BL/6 × DBA2 F1) female mice crossed with males of the same strain at 0.5 dpc. For Cas9-encoding mRNA and sgRNA, those RNAs were coinjected into the cytoplasm. Injected eggs were cultured overnight in M16 medium (Sigma) at 37°C with 5% CO_2_. Resulting two-cell embryos were transferred into the oviducts of pseudo-pregnant MCH/ICR females.

### Analysis of mutations by Surveyor nucleases

To detect Fgf10 TALEN- and CRISPR-induced mutations in F0 embryos, genomic DNA was extracted from the tail tips of fetuses and newborns. Targeted genomic loci were amplified by using the KOD-Plus-Neo (TOYOBO, Osaka, Japan) DNA polymerase with 3′ to 5′ proofreading exonuclease activity, according to the manufacturer's instructions. Primers were designed to anneal ~200 to 250 bp upstream and downstream from the expected cut sites. PCR was carried out with the following oligonucleotides: Ex1F (5′-CAGCAGGTCT TACCCTTCCA-3′) and Ex1R (5′-TACAGGGGTT GGGGACATAA-3′) were used to amplify a 521-bp fragment surrounding the target site in Exon1. Ex3F (5′-TGACTCTTCT GTTGTTAGCGT TG-3′) and Ex3R (5′-ACATCCAAAG CCTTCCTTCC-3′) were used to amplify a 501-bp fragment surrounding the target site in Exon3.

To allow complementary but mismatched strands to anneal, PCR products (400 ng), purified by the Wizard SV Gel and PCR Clean-up System (Promega), were incubated at 95°C for 5 min in 10 mM Tris-HCl (pH 8.5), 75 mM KCl and 1.5 mM MgCl_2_. Nuclease S (0.4 μl) and Enhancer S (0.4 μl) (Surveyor Mutation Detection Kit, Transgenomic, Omaha, NE) were added. Samples were incubated at 42°C for 30 min to digest the annealed PCR products at the mismatched sites. Nuclease S-digested PCR products were analysed by agarose gel electrophoresis.

### DNA sequencing of mutated endogenous gene target sites

The purified PCR products were used for either direct or cloned sequencing. For the DNA sequencing analysis of each allele induced by the TALEN or CRISPR/Cas system, 3′-terminal ‘A' overhangs were added to the purified blunt-ended PCR amplicons and subcloned into a plasmid using the DynaExpress TA PCR Cloning Kit (BioDynamics Laboratory, Tokyo, Japan). After transformation of these reactions, plasmids were extracted from the resultant *Escherichia coli* colonies. Sequencing was performed with the BigDye Terminator Cycle Sequencing Kit and an ABI 3500 Genetic Analyser (Applied Biosystems, Foster City, CA). Mutated alleles were identified by comparison to the wild-type sequence.

## Author Contributions

A.Y. and S.N. designed the work. A.Y., S.M. and T.W. performed the experiments. A.Y., S.M., T.S., S.O., T.Y., S.N., T.M. and E.T. analysed the data. A.Y. wrote the paper. All co-authors contributed in the form of discussion and critical comments.

## Supplementary Material

Supplementary Information Supplementary Figure S1

## Figures and Tables

**Table 1:** TALEN-mediated Fgf10 gene targeting in BDF1 mice

Injection/route	Dose (ng/μl)	Injected/transferred (%)	No. (%) embryos	Newborns, M, F (%)	NHEJ events (%)*	Limb defects (%)
mRNA/IC	50	142/93 (65)	-	17, 15 (34)	15 (47)	-
mRNA/IC	50	105/66 (63)	27 (41)	-	13 (48)	1 (8)
mRNA/IC	20	116/79 (68)	43 (54)	-	11 (26)	1 (9)
mRNA/IC	10	159/107 (67)	59 (55)	-	16 (27)	0 (0)
mRNA/PNI	20	16/13 (81)	8 (62)	-	1 (13)	0 (0)
mRNA/PNI	10	114/72 (63)	19 (26)	-	4 (21)	0 (0)

Embryos were isolated between embryonic day 13.5 and 18.5. *Determined by both Cel-I endonuclease assay and direct sequencing. IC, intracytoplasmic; PNI, pronuclear injection.

**Table 2:** Germline transmission of TALEN-induced alleles and production of limb-defect knockout embryos

Parents' genotypes		No. embryos	F1 genotypes (No. embryos)	No. limb defects
Male	Female			
WT/−1/−3 (M3)	WT/WT	11	WT/WT (3), −1/WT (5), −3/WT (3)	0
WT/+1/−4/−9 (M11)	WT/WT	12	WT/WT (1), +1/WT (6), −4/WT (1), −9/WT (4)	0
WT/+1/−9 (M1)	WT/−30+2 (F11)	7	WT/WT (2), WT/−30+2 (2), +1/WT (1), −9/WT (1), −9/−30+2 (1)	0
WT/−9 (M15)	WT/−24 (F3)	7	WT/WT (1), −9/WT (4), WT/−24 (2), −9/−24 (0)	0
WT/−5 (M12)	WT/−1 (F1 gen of M3)	11	-	4
WT/−1 (F1 gen of M3)	WT/−1 (F1 gen of M3)	12	-	6

F1 alleles were determined by sequencing. Genotyping was not performed for the bottom two rows, in which embryos with limb defects were obtained. gen, generation.

**Table 3:** CRISPR/Cas-mediated Fgf10 gene targeting in BDF1 mice

Target region	Cas9/sgRNA (ng/μl)	No. injected/transferred (%)	No. (%) embryos	ED	NHEJ events (%)*	No. (%) limb defects
#143	10/1	52/22 (42)	15 (68)	16.5	15/15 (100)	8 (53)
	10/0.1	27/17 (63)	10 (59)	16.5	9/10 (90)	6 (60)
#547	10/1	92/28 (30)	4 (14)	14.5	-	4 (100)
	10/0.1	58/35 (60)	14 (40)	17.0	-	13 (93)
#563	10/1	67/24 (36)	11 (46)	14.5	-	10 (91)
	10/0.1	50/21 (35)	20 (99)	16.5	-	8 (40)

Cas9 mRNA and sgRNA were injected into the cytoplasm at the indicated concentrations. Embryos were isolated between embryonic day (ED) 14.5 and 17.0. *Determined by both Cel-I endonuclease assay and direct sequencing. Surveyor (Cel-I) endonuclease assay was not performed for embryos injected with CRSPR_547 and 563.
