# Supplementary material for: Highly efficient targeted mutagenesis in one-cell mouse embryos mediated by the TALEN and CRISPR/Cas systems
Source: Sci Rep. 2014 Jul 16;4:5705. doi: 10.1038/srep05705 (PMC4099983; doi:10.1038/srep05705)
Supplement: Supplementary Information — Supplementary Figure S1 [file srep05705-s1.pdf]

Supplementary Information

Highly efficient targeted mutagenesis in one-cell mouse embryos mediated by the TALEN and CRISPR/Cas systems

Akihiro Yasue[1,*], Silvia Naomi Mitsui[1], Takahito Watanabe[2], Tetsushi Sakuma[3], Seiichi Oyadomari[4], Takashi Yamamoto[3], Sumihare Noji[2], Taro Mito[2] & Eiji Tanaka[1]

[1]Department of Orthodontics and Dentofacial Orthopedics, Institute of Health Biosciences, The University of Tokushima Graduate School, 3-18-15 Kuramoto-cho, Tokushima 770-8504, Japan, [2]Department of Life Systems, Institute of Technology and Science, The University of Tokushima, 2-1 Minami-Jyosanjima-cho, Tokushima 770-8506, Japan, [3]Department of Mathematical and Life Sciences, Graduate School of Science, Hiroshima University, 1-3-1 Kagamiyama, Higashi-Hiroshima, Hiroshima 739-8526, Japan, [4]Division of Molecular Biology, Institute for Genome Research, The University of Tokushima, 3-18-15 Kuramoto-cho, Tokushima 770-8503, Japan.

547-#10

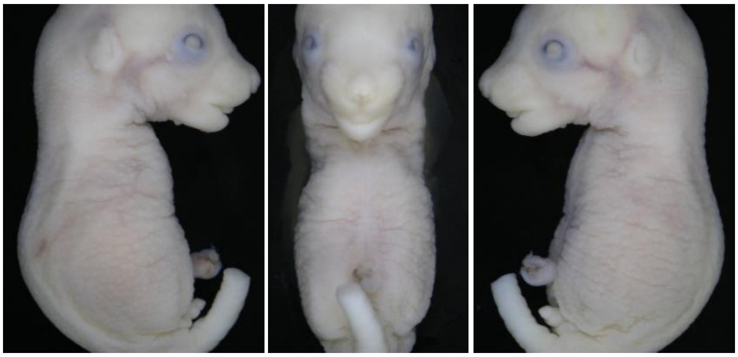

547-#12

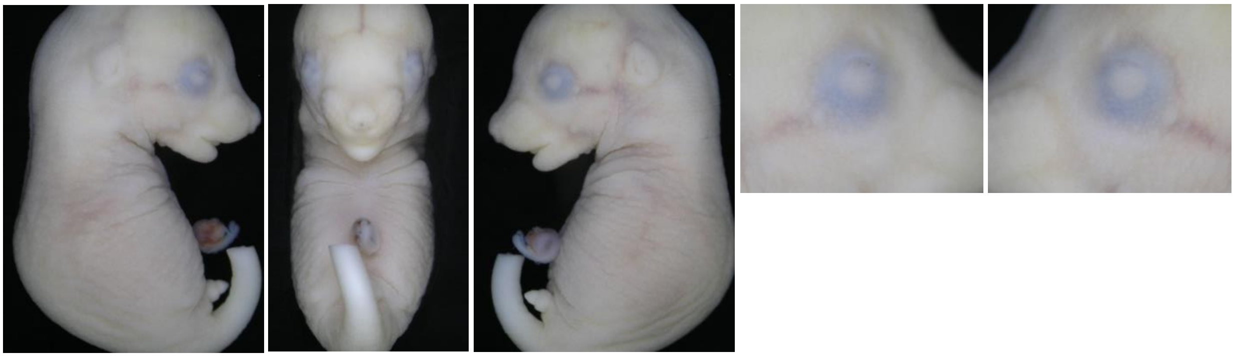

563-#17

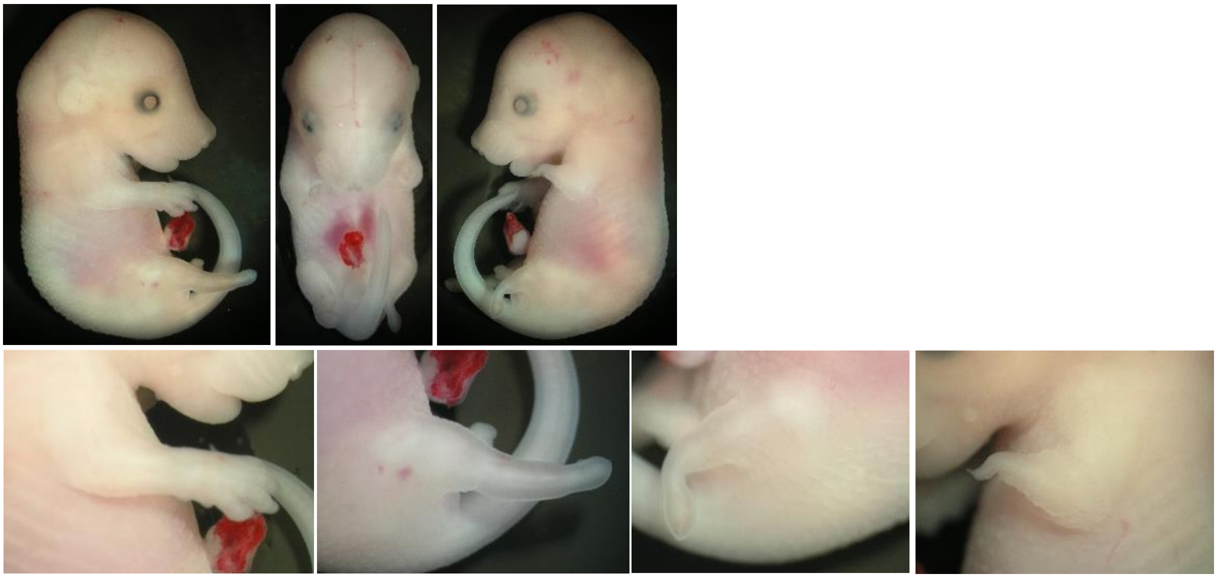

563-#19

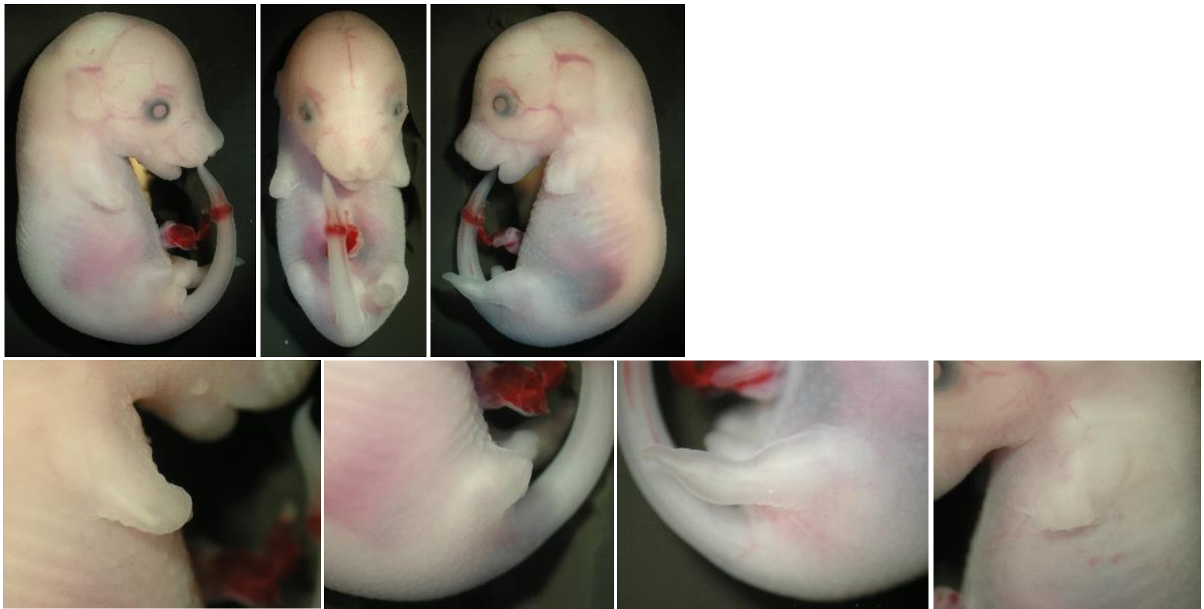

Supplementary Figure S1: Examples of embryos produced by CRISPR_547 and 563. Examples of embryos with limb defects are shown in the magnified panels for eyes or limbs (547-#10 and #12 at E17.0, 563-#17 and #19 at E16.5). Embryos 547-#10 and #12 show complete limb deficiency; however, #12 has eyelids. For embryos 563-#17 and #19, incomplete limb-deficiency phenotypes are shown in the magnified panels for all limbs.
